# The Pyrazole Scaffold in Anticancer Drug Discovery: A Review of Synthetic Approaches, Structure–Activity Relationships, and Target-Based Mechanism of Action

**DOI:** 10.3390/ijms27083403

**Published:** 2026-04-10

**Authors:** Krishnapriya K R, Veda B. Hacholli, Marcin Gackowski, Dariusz Maciej Pisklak, Abhishek Kumar, Łukasz Szeleszczuk

**Affiliations:** 1 Department of Pharmaceutical Chemistry, NGSM Institute of Pharmaceutical Sciences (NGSMIPS), Nitte (Deemed to be University), Mangalore 575018, Karnataka, India; krishnapriyakr@yenepoya.edu.in; 2 Department of Pharmaceutical Chemistry, Yenepoya Pharmacy College & Research Centre, Yenepoya (Deemed to be University), Mangalore 575018, Karnataka, India; 3 Department of Pharmaceutical Chemistry, Faculty of Pharmacy, Nitte College of Pharmaceutical Sciences, Nitte (Deemed to be University), Bengaluru 560064, Karnataka, India; vedab.hacholli@nitte.edu.in; 4 Department of Toxicology and Bromatology, Faculty of Pharmacy, L. Rydygier Collegium Medicum in Bydgoszcz, Nicolaus Copernicus University in Torun, A. Jurasza 2 Street, 85089 Bydgoszcz, Poland; 5 Department of Organic and Physical Chemistry, Faculty of Pharmacy, Medical University of Warsaw, Banacha 1 Street, 02093 Warsaw, Polandlukasz.szeleszczuk@wum.edu.pl (Ł.S.)

**Keywords:** pyrazole derivatives, anticancer agents, structure–activity relationship, medicinal chemistry, molecular targets

## Abstract

Pyrazole derivatives have emerged as an important class of heterocyclic compounds in anticancer research due to their structural versatility and broad spectrum of biological activities. This review provides a concise overview of recent advances in the development of pyrazole-based anticancer agents, with emphasis on synthetic strategies, structure–activity relationships, and molecular mechanisms of action. Common synthetic approaches, particularly condensation and cyclization reactions, have enabled the preparation of structurally diverse pyrazole derivatives for biological evaluation. Available evidence indicates that the type and position of substituents within the pyrazole scaffold markedly influence anticancer potency, selectivity, and target affinity. Reported compounds act through multiple mechanisms, including inhibition of cancer-related targets such as tubulin, epidermal growth factor receptor (EGFR), cyclin-dependent kinases (CDKs), Bruton tyrosine kinase (BTK), and deoxyribonucleic acid (DNA)-associated pathways, as well as induction of apoptosis and disruption of cell-cycle progression. Several pyrazole derivatives have shown promising activity in in vitro and in vivo models. Overall, the findings summarized in this review identify the pyrazole scaffold as a valuable platform for the design and optimization of novel anticancer agents and support its continued exploration in medicinal chemistry.

## 1. Introduction

Ranked as the second most common cause of death worldwide, cancer remains a significant threat to human health. The International Agency for Research on Cancer (IARC) 2022 report documented a global incidence of approximately 20 million newly diagnosed malignancies and 9.7 million cancer-related mortalities, with breast and pulmonary carcinomas representing the most frequently occurring tumor types [1]. Cancer has emerged as one of the leading causes of death globally in the 21st century, with millions of deaths attributable to malignancies of the breast, brain, bladder, prostate, non-small cell lung, kidney, colorectal, oral, oropharyngeal, and skin. “Cancer is a disease where certain cells in the body grow out of control and invade other areas of the body”. A cluster of rapidly proliferating cells that can form a widely dispersed lump or mass is called a tumor or neoplasm. Disease development can result from a multitude of factors, including genetic mutations, environmental influences, lifestyle choices, and infections (caused by bacteria, viruses, or parasites) [2,3,4]. Currently, the primary cancer treatment approaches include immunotherapy, genetic therapy, and targeted molecular therapy. Chemotherapy is one of the most successful cancer treatment methods available. Despite the development of numerous targeted anticancer medicines, the main reasons for clinical use failure are significant adverse events and drug resistance. Thus, finding and creating potent anti-tumor medications remains a challenging task [5,6].

Pyrazole is a heterocyclic compound characterized by a five-membered ring containing three carbon and two nitrogen atoms. It was first introduced by Ludwig Knorr in 1883, and in 1959, 1-pyrazolyl-alanine became the first naturally discovered pyrazole, isolated from watermelon seeds [7]. This ring structure serves as a valuable scaffold for designing compounds with a variety of biological roles [8,9], including antibacterial and antifungal [10,11], antitumor [12,13], anti-inflammatory [14,15] and analgesic [16], antitubercular [17,18], antiviral [19,20], anti-Alzheimer’s [21,22], α-glycosidase inhibitory [23], anti-diabetic [24,25], antileishmanial [26,27], anti-malarial [28,29], antidepressant [30], antihypertensive [31,32], anti-arthritic [33] activities. The anticancer potential of pyrazole was first recognized in the 1970s. Since then, numerous studies have investigated the synthesis, structure–activity relationships, and biological assessment of pyrazole derivatives as anticancer compounds [34]. These studies have shown that pyrazole derivatives can inhibit various kinases, induce apoptosis, and disrupt cell-cycle progression, thereby suppressing cancer cell growth and proliferation [35,36].

Advantages of pyrazole as an anticancer agent

- High potency and selectivity against specific cancer cell lines [37]
- Ability to inhibit multiple kinases and signaling pathways [37,38,39,40]
- Potential for oral administration and good pharmacokinetic profiles [38,39,40]
- Low toxicity and side effects compared to traditional chemotherapy agents [37]

**Figure 1:**
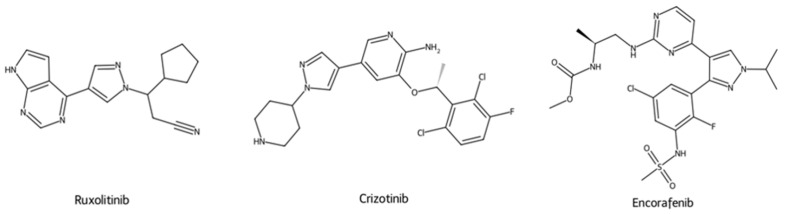
Examples of marketed anticancer drugs containing the pyrazole scaffold.

The pyrazole framework serves as the central scaffold of many leading anticancer drugs, contributing significantly to their biological activity and therapeutic efficacy. As shown in Figure 1, several clinically relevant anticancer agents incorporate the pyrazole scaffold, which enhances target selectivity and pharmacological performance through its heterocyclic architecture.

Representative marketed examples further illustrate the translational relevance of the pyrazole scaffold. Ruxolitinib, crizotinib, and encorafenib demonstrate that pyrazole-containing frameworks can be successfully incorporated into clinically used anticancer agents acting through distinct kinase-directed mechanisms, including Janus kinase (JAK) inhibition, inhibition of anaplastic lymphoma kinase (ALK), c-ros oncogene 1 (ROS1), and mesenchymal–epithelial transition factor (MET), as well as inhibition of B-Raf proto-oncogene serine/threonine kinase (BRAF), respectively [38,39,40].

Recent literature reviews have addressed important aspects of pyrazole chemistry and pharmacology, including the role of the pyrazole scaffold in kinase-targeted anticancer design, general synthetic approaches to pyrazole derivatives, their broad biological significance, and recent advances in pyrazole-based anticancer agents. Very recent reviews published in 2025–2026 have further expanded this landscape by emphasizing updated synthetic strategies, hybrid pyrazole systems, and structure–activity relationship trends relevant to anticancer drug discovery [41,42,43,44,45,46,47]. However, these contributions differ in scope, often emphasizing either synthetic methodology or broader pharmacological coverage. In contrast, the present review is specifically focused on the pyrazole scaffold in anticancer drug discovery and integrates synthetic approaches, structure–activity relationships, and target-based mechanisms of action within a single oncology-oriented framework.

This review provides an integrated overview of the anticancer potential of pyrazole derivatives, with particular emphasis on structural features, mechanisms of action, and the available preclinical and clinical evidence. It focuses on pyrazole-based compounds with documented anticancer activity, their interactions with relevant molecular targets and signaling pathways, and the main challenges and future directions associated with their development. By organizing the available literature and identifying the most informative trends, the review aims to serve as a practical resource for researchers and clinicians interested in the therapeutic potential of pyrazoles in oncology.

## 2. Chemistry and Structure–Activity Relationships of Pyrazoles

**Figure 2:**
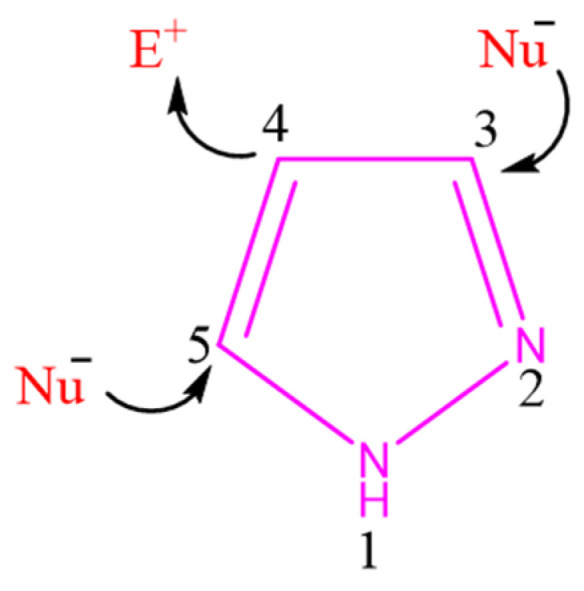
Chemical structure of pyrazole.

Pyrazole is a five-membered heterocyclic compound containing two adjacent nitrogen atoms, which contribute to its unique electronic configuration and chemical stability. As shown in Figure 2, aromaticity is maintained by a six-π-electron system formed by the two double bonds in the ring and the lone pair of the pyrrole-like nitrogen, whereas the pyridine-like nitrogen remains sp^2^-hybridized and does not contribute its lone pair to the aromatic sextet. This electronic distribution also gives rise to differentiated reactivity within the ring, with the C3 and C5 positions showing nucleophilic character and the C4 position being relatively electrophilic. Such polarization helps explain the characteristic substitution pattern of pyrazole and its value as a versatile heterocyclic synthon in medicinal chemistry [48,49].

Substitutions on the pyrazole ring significantly impact their chemical and biological properties. N-unsubstituted pyrazole derivatives have amphoteric capabilities and can contribute to and receive hydrogen bonds simultaneously. However, substituting the pyrrole-like nitrogen destroys the heterocycle’s acidic character and hydrogen bond capacity. Pyrazole-containing drugs are more durable against oxygenases and resistant to oxidative metabolism [50,51,52]. More broadly, the relationship between pyrazole substitution patterns and biological activity has been discussed in previous reports [53,54].

## 3. Synthesis of Pyrazole-Based Derivatives

**Figure 3:**
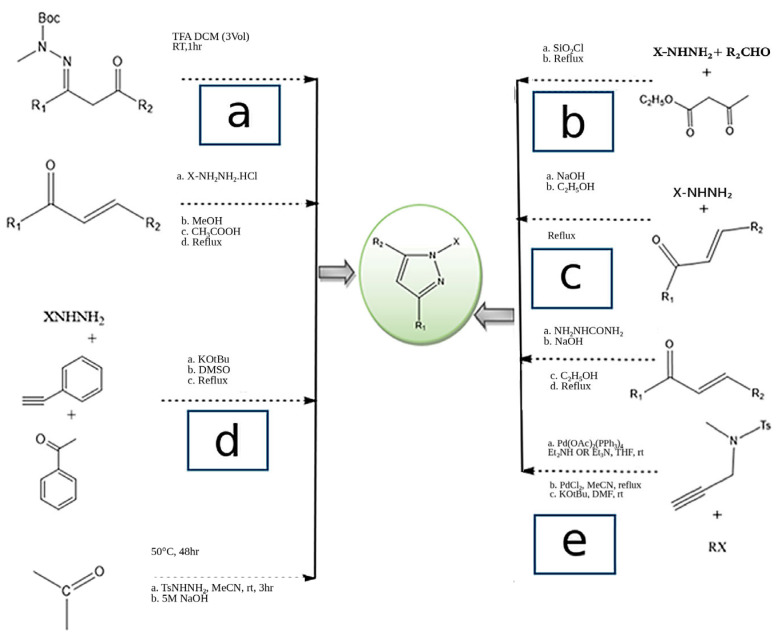
General methods of synthesis of pyrazole derivatives. Representative route clusters are indicated as (**a**–**e**).

Representative route clusters discussed in this section are summarized in Figure 3 and indicated as 3a–3e, highlighting classical precursor-based assembly (Figure 3a), cyclization of α,β-unsaturated carbonyl systems with hydrazine derivatives (Figure 3b,c), transition-metal-enabled annulation/coupling chemistry within the broader synthetic overview, and carbonyl-based Knoevenagel–Fischer/Michael-type pathways (Figure 3e).

### 3.1. Classical Methods for Pyrazole Synthesis

Traditional synthetic approaches continue to play a central role in the construction of the pyrazole nucleus. Among these, the Knorr synthesis is one of the most widely used methods, involving the condensation of hydrazine or substituted hydrazines with 1,3-dicarbonyl compounds. This strategy is appreciated for its simplicity and adaptability, allowing the introduction of diverse substituents at different positions of the pyrazole ring. In addition, [3+2] cycloaddition reactions, often described as Pechmann-type syntheses, employ 1,3-dipolar intermediates, such as diazo compounds or nitrile imines, to form pyrazoles via reactions with alkenes or alkynes. These reactions typically proceed under mild conditions and offer good regioselectivity [35,49,55]. A representative classical precursor-based route of this type is shown in Figure 3a.

### 3.2. Role of Transition-Metal-Catalyzed Approaches

Advances in transition-metal catalysis have significantly expanded the synthetic options for pyrazole derivatives. Palladium-catalyzed coupling and annulation reactions have emerged as powerful one-pot methodologies for the synthesis of substituted pyrazoles. For example, the reaction of N-tosyl-N-propargylhydrazine with aryl iodides or vinyl triflates enables the formation of 3-aryl or vinyl-substituted 1*H*-pyrazoles [56]. These methods are particularly useful due to their tolerance toward a wide range of functional groups, allowing fine modulation of electronic and pharmacological properties [55]. A representative transition-metal-catalyzed annulation/coupling route is also reflected in the broader synthetic overview presented in Figure 3.

### 3.3. Diazo-Based and Cycloaddition Strategies

Diazo chemistry has also contributed significantly to the diversification of pyrazole synthesis. Diazo intermediates generated in situ from aldehyde tosylhydrazones readily participate in 1,3-dipolar cycloaddition reactions with acetylene equivalents, such as N-vinylimidazole. These reactions provide regioselectively substituted pyrazoles and are valued for their functional group compatibility and operational convenience, making them attractive for medicinal chemistry applications [49,55]. Related cycloaddition-oriented strategies are exemplified in Figure 3d.

### 3.4. Condensation and Chalcone-Based Routes

Condensation reactions remain popular because of their operational simplicity and efficiency. The Claisen–Schmidt condensation is commonly employed to prepare α,β-unsaturated intermediates from 1,3-dicarbonyl compounds, which can subsequently cyclize with hydrazines to yield pyrazolines or aromatized pyrazoles [57,58,59]. Similarly, the cyclocondensation of chalcones with hydrazine hydrate under acidic conditions is widely used, particularly because it can be adapted to microwave-assisted or solvent-free conditions to enhance reaction rates and yields. Typical cyclizations of α,β-unsaturated carbonyl systems with hydrazine derivatives are illustrated in Figure 3b,c.

### 3.5. Knoevenagel–Fischer and Michael Addition Pathways

Additional synthetic routes include the Knoevenagel–Fischer synthesis, in which α,β-unsaturated carbonyl compounds react with hydrazine derivatives in glacial acetic acid to undergo intramolecular cyclization. Michael addition reactions between chalcones and hydrazines under basic conditions, such as triethylamine-mediated cyclization, also provide efficient access to substituted pyrazolines, which can be further oxidized to form pyrazoles [57,60,61]. Representative carbonyl-based pathways of this type are illustrated in Figure 3e.

## 4. Structure–Activity Relationships of Pyrazole Derivatives

Understanding the structure–activity relationships (SARs) of pyrazole derivatives is essential for enhancing their pharmacological profiles and optimizing their efficacy as anticancer agents. The pyrazole scaffold, characterized by a five-membered heterocyclic ring, offers a versatile template for modifying electronic, steric, and hydrophobic properties, influencing biological activity. A thorough understanding of the SARs that govern pyrazole derivatives will aid the strategic design and synthesis of new analogs with improved effectiveness and selectivity, thereby accelerating the development of pyrazole-based anticancer treatments [44,62].

General structure–activity trends in pyrazole-based anticancer agents should be interpreted with caution, because many reported observations are derived from spe-cific compound series rather than from universally applicable SAR rules. In several pyrazole series, electron-withdrawing substituents, lipophilic aryl groups, and appropriately positioned heterocyclic or linker fragments have been associated with improved antiproliferative activity; however, these effects are strongly dependent on the overall scaffold, substitution pattern, and biological model used for evaluation [63,64,65,66,67,68,69,70,71,72,73,74,75]. Likewise, the influence of steric bulk, lipophilicity, and hydrogen-bonding functionality is context-dependent and may vary across different pyrazole chemotypes. Molecular modeling and docking studies have provided useful mechanistic insights into pyrazole–target interactions, but such findings should be interpreted as supportive rather than universally predictive in the absence of broader experimental validation [64,65,66,68,69,70,71,72,73].

**Figure 4:**
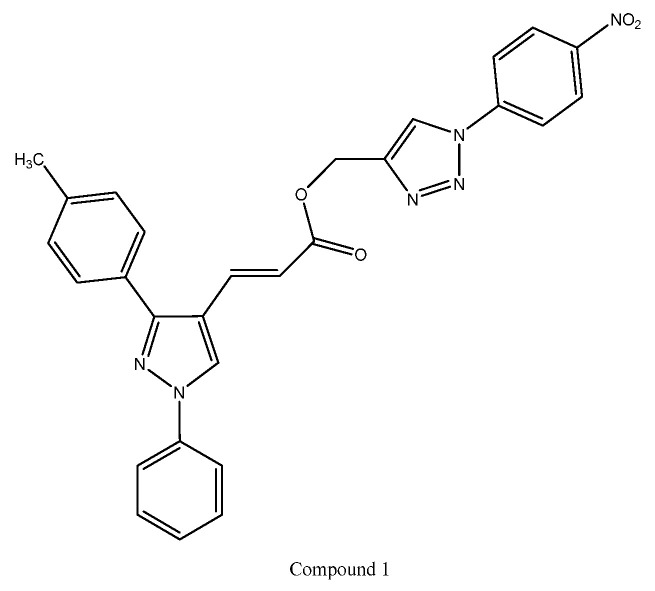
Structure of compound **1** representing a 1,2,3-triazole-linked 3-(1,3-diphenyl-1*H*-pyrazol-4-yl)acrylate derivative.

A series of 3-(1,3-diphenyl-1*H*-pyrazol-4-yl)acrylate derivatives incorporating 1,2,3-triazole linkers was designed and synthesized through a triazole-mediated hybridization strategy to enhance anticancer potential. The synthesized compounds were biologically evaluated for in vitro anticancer activity using the sulforhodamine B (SRB) assay against four human cancer cell lines: MCF-7 (breast), A549 (lung), HCT-116, and HT-29 (colon). Several derivatives demonstrated significant growth inhibition across the tested cell lines. Structure–activity relationship (SAR) analysis revealed that electron-donating substituents such as methyl and methoxy groups at the R-position of the diphenyl pyrazole core markedly improved anticancer activity, whereas unsubstituted analogues showed comparatively weaker effects. In contrast, the introduction of electron-withdrawing groups (fluoro and chloro) at the same position diminished potency; however, when these groups were present on the phenyl ring attached to the triazole moiety, an enhancement in anticancer activity was observed. Among the evaluated derivatives, Compound 1 emerged as the most potent analogue, exhibiting IC_50_ values of 1.962 μM (A549), 3.597 μM (HCT-116), 1.764 μM (MCF-7), and 4.496 μM (HT-29). As shown in Figure 4, the enhanced cytotoxic activity of Compound 1 may be attributed to the presence of a 1,2,3-triazole linker and an electron-withdrawing nitro-substituted phenyl ring, which improve molecular stability and facilitate stronger interactions with biological targets [63].

**Figure 5:**
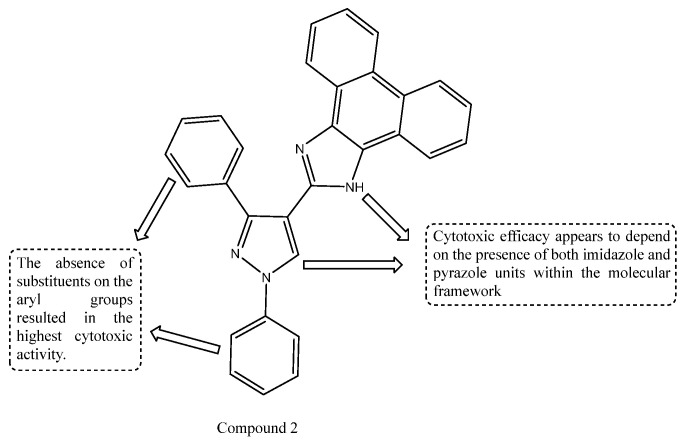
Structure of compound **2** representing a pyrazolyl phenanthroimidazole derivative.

A series of novel pyrazolyl-phenanthroimidazole derivatives was synthesized via a multistep synthetic strategy and fully characterized using standard physical and spectroscopic techniques. The synthesized compounds were evaluated for cytotoxicity against pancreatic cancer cell lines AsPC-1 and SW1990, as well as against normal MRC-5 fibroblast cells. Several derivatives exhibited promising anticancer activity, while maintaining moderate toxicity toward normal cells. Structure–activity relationship (SAR) analysis indicated that the absence of aryl substituents on the imidazole–pyrazole core significantly enhanced cytotoxic activity. Among the series, Compound 2 emerged as the most active analogue, showing IC_50_ values of 30.3 ± 0.45 µM (AsPC-1) and 32.4 ± 0.65 µM (SW1990), with moderate toxicity toward MRC-5 cells (IC_50_ = 55.5 ± 3.50 µM). As shown in Figure 5, the enhanced cytotoxic activity may be attributed to the synergistic presence of imidazole and pyrazole moieties within the molecular framework, along with the absence of substituents on the aryl group, which enhances the compound’s biological efficacy [64].

**Figure 6:**
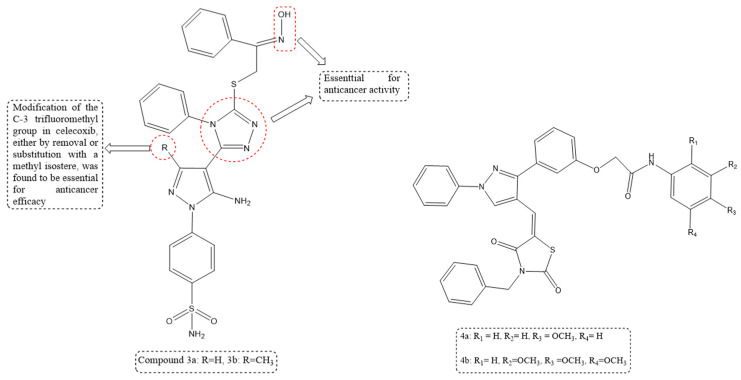
Structures of compounds **3a** and **3b** representing 1,2,4-triazole–pyrazole derivatives and compounds **4a** and **4b** representing (Z)-2-(3-(4-((3-benzyl-2,4-dioxothiazolidin-5-ylidene)methyl)-1-phenyl-1*H*-pyrazol-3-yl)phenoxy)-N-arylacetamide derivatives.

Two new series of pyrazole–1,2,4-triazole hybrid molecules were rationally designed and synthesized through a hybridization strategy inspired by the celecoxib pharmacophore. The synthesized compounds were evaluated for their in vitro anticancer activity against A549 (lung), MCF-7 (breast), HCT-116 (colon), and PC-3 (prostate) cancer cell lines, and most derivatives exhibited strong cytotoxicity. Structure–activity relationship (SAR) analysis highlighted that introduction of a 1,2,4-triazole linker at the C-4 position of the pyrazole ring, replacement or removal of the C-3 trifluoromethyl group with a methyl isostere, and attachment of an oxime NO-donor moiety at the triazole-linked sulfur significantly enhanced anticancer potency and selectivity. Additionally, compounds **3a** and **3b** induced apoptosis, inhibited cell proliferation, and arrested the cell cycle in PC-3 cells. Among the synthesized hybrids, sulphamoyl-containing derivatives **3a** and **3b** emerged as the most active compounds, exhibiting IC_50_ values of 5.34 and 6.48 μM (A549), 4.71 and 5.33 μM (MCF-7), 4.39 and 5.12 μM (HCT-116), and 3.66 and 4.37 μM (PC-3), respectively. As shown in Figure 6, the enhanced anticancer activity of compounds **3a** and **3b** may be attributed to the presence of the sulphamoyl functional group, which enhances binding to biological targets and increases cytotoxicity against cancer cell lines [65].

A series of (Z)-2-(3-(4-((3-benzyl-2,4-dioxothiazolidin-5-ylidene)methyl)-1-phenyl-1*H*-pyrazol-3-yl)phenoxy)-N-arylacetamide derivatives were synthesized through efficient multistep synthetic procedures, affording good yields, and were structurally characterized using ^1^H NMR, ^13^C NMR, IR spectroscopy, and ESI–MS techniques. The synthesized compounds were evaluated for their in vitro cytotoxic activity against MCF-7 human breast cancer cells over a concentration range of 0.625–10 µM. Several derivatives demonstrated potent anticancer activity, with structure–activity relationship (SAR) analysis revealing that N-aryl acetamide derivatives incorporating heterocyclic cores exhibited enhanced biological activity, primarily due to the amide linkage, which played a crucial role in activity modulation. Substituent variations at positions R_1_, R_2_, R_3_, and R_4_ significantly influenced lipophilicity, while the pyrazole and 2,4-thiazolidinedione moieties served as essential drug-like scaffolds. Additionally, incorporation of the amide group improved polarity and solubility, thereby reducing molecular aggregation. Among the synthesized series, Compounds **4a** and **4b** emerged as the most potent analogues, surpassing cisplatin in activity and exhibiting IC_50_ values of 0.604 µM and 0.665 µM, respectively. As shown in Figure 6, the enhanced anticancer activity may be attributed to the presence of electron-donating methoxy substituents, such as *para*-methoxy, 3,4-dimethoxy, and 3,4,5-trimethoxy groups, which enhance the compounds’ interactions with biological targets and thereby increase cytotoxicity [66].

**Figure 7:**
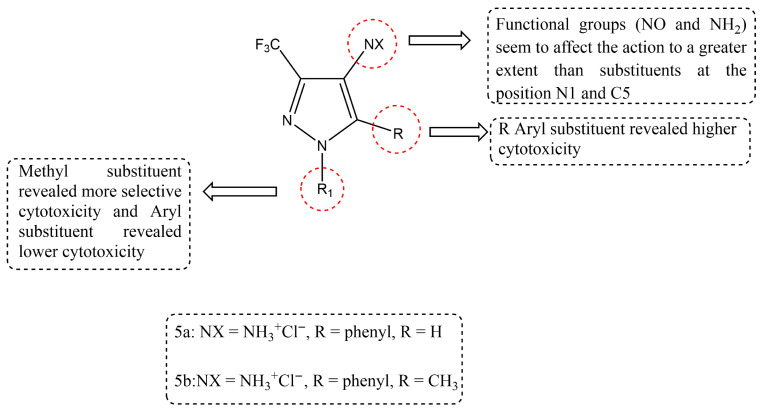
Chemical structure of compounds **5a** and **5b** representing 4-aminopyrazole derivatives.

A new set of 4-aminopyrazole derivatives was designed and synthesized through a systematic structural modification approach to explore their anticancer potential. The synthesized compounds were evaluated for their in vitro cytotoxicity against HeLa cells and human dermal fibroblasts, using camptothecin as the reference standard. Several derivatives demonstrated significant cytotoxicity, with structure–activity relationship (SAR) analysis revealing that substituent variations at all three scaffold positions markedly influenced biological activity. In particular, modifications at the R and R^1^ positions were critical for fine-tuning cytotoxic potency, selectivity, and overall anticancer efficacy. Among the evaluated series, Compound **5b** emerged as the most potent analogue, with an IC_50_ of 0.074 ± 0.005 µM, while Compound **5a** also showed notable activity (IC_50_ = 1.86 ± 0.12 µM), both outperforming the reference drug camptothecin (IC_50_ = 1.66 ± 0.97 µM). As shown in Figure 7, the enhanced cytotoxic activity of these derivatives may be attributed to the presence of specific functional groups such as NO and NH_2_ at the N1 and C5 positions, along with methyl substitution, which significantly influence the biological activity of 4-aminopyrazole derivatives [67].

**Figure 8:**
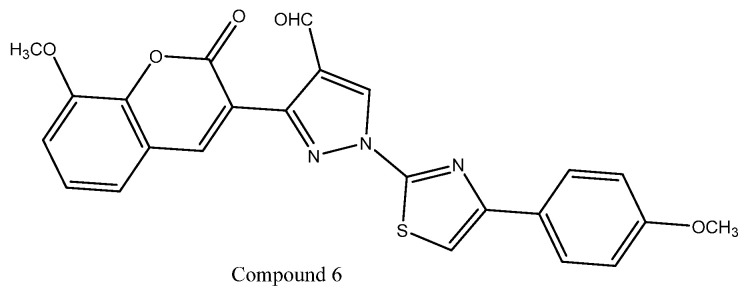
Chemical structure of compound **6** representing thiazolyl–pyrazole carbaldehyde hybrids.

A series of thiazolyl–pyrazole carbaldehyde hybrid compounds was synthesized via a systematic synthetic approach and evaluated for in vitro anticancer activity. The synthesized derivatives were screened for antiproliferative effects against HeLa, MCF-7, and A549 cancer cell lines using nocodazole as the reference standard. Most compounds exhibited mild to weak inhibitory activity, particularly against HeLa cells; however, structure–activity relationship (SAR) analysis revealed that the presence of additional methoxy substituents at the R_1_, R_2_, and R_3_ positions significantly enhanced anticancer potency. Furthermore, incorporation of a δ-lactone ring into the coumarin moiety was identified as a critical structural feature contributing to improved biological activity, whereas replacing the coumarin oxygen atom with an NH group led to a marked reduction in activity. Among the synthesized series, compound **6** emerged as the most potent analogue, exhibiting IC_50_ values of 9.05 ± 0.04 µM (HeLa), 7.12 ± 0.04 µM (MCF-7), and 6.34 ± 0.06 µM (A549). As shown in Figure 8, the presence of key functional groups in compound **6** may contribute to its enhanced cytotoxic activity against the tested cancer cell lines [68].

**Figure 9:**
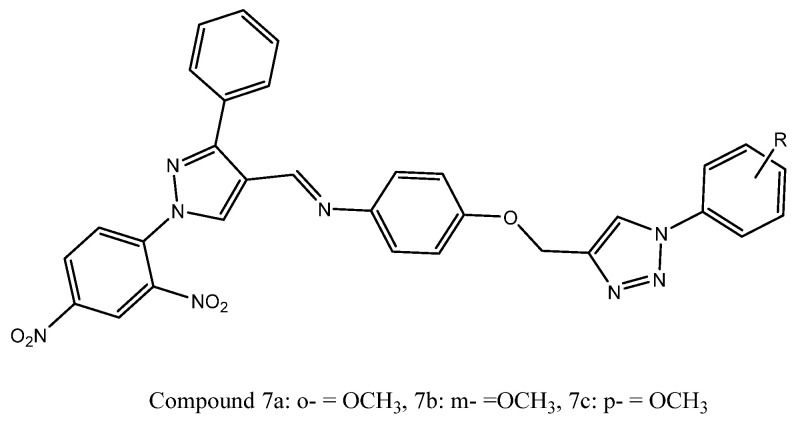
Chemical structure of compounds **7a**–**7c** representing dinitrophenyl-pyrazole-linked triazole derivatives.

A new series of dinitrophenylpyrazole-linked triazole derivatives was rationally designed and synthesized to explore their anticancer potential. The synthesized compounds were evaluated for in vitro cytotoxicity against MCF-7, HeLa, and Caco-2 cancer cell lines using the MTT assay, with combretastatin-A4 as the reference drug. Several derivatives demonstrated significant cytotoxic effects, particularly against HeLa cells. Structure–activity relationship (SAR) analysis revealed that the presence of methoxy substituents at ortho, meta, or para positions on the phenyl ring markedly enhanced anticancer potency. In contrast, electron-withdrawing substituents, such as chloro and bromo groups, reduced cytotoxic activity. Among the synthesized series, compounds **7a**, **7b**, and **7c** emerged as the most active analogues, exhibiting IC_50_ values of 4.0, 5.0, and 6.0 µM against HeLa cells, respectively, outperforming combretastatin-A4 (IC_50_ = 9.0 µM), while compound **7c** also showed notable activity against MCF-7 cells with an IC_50_ value of 8.0 µM. As shown in Figure 9, variations in the methoxy (–OCH_3_) substitution at the ortho, meta, and para positions influence the cytotoxic activity of the synthesized derivatives [69].

**Figure 10:**
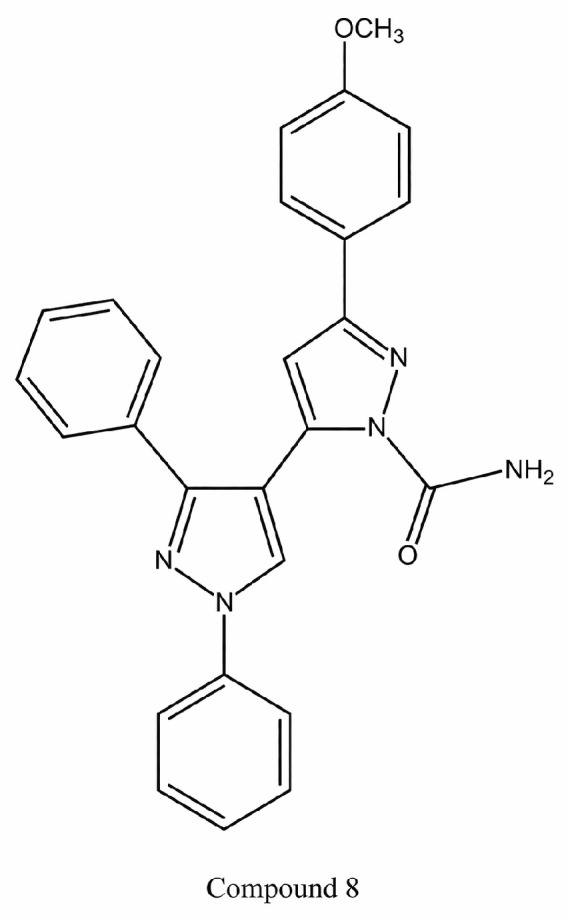
Structure of compound **8** representing pyrazole–pyrazoline derivatives.

A novel series of pyrazole–pyrazoline hybrid compounds was designed and synthesized to investigate their anticancer potential. The synthesized derivatives were evaluated for in vitro cytotoxicity using the MTT assay against MCF-7, A549, SiHa, COLO205, and HepG2 cancer cell lines, with 5-fluorouracil as the reference standard. Several compounds exhibited significant anticancer activity across multiple cell lines, with structure–activity relationship (SAR) analysis revealing that the absence of substituents on ring B was critical for enhanced potency. In contrast, increased electronegativity at this position led to reduced activity. Furthermore, SAR evaluation of ring C (R^1^ position) indicated that methoxy substitution markedly improved anticancer activity, whereas electron-withdrawing substituents diminished efficacy. Among the evaluated series, compound **8** emerged as the most potent analogue, displaying IC_50_ values of 4.94 µM (A549), 4.54 µM (SiHa), 4.86 µM (COLO205), and 2.09 µM (HepG2), along with minimal toxicity toward normal HaCaT cells (IC_50_ > 50 µM). As shown in Figure 10, the presence of substituted aromatic rings and amide functional groups may contribute to its enhanced anticancer activity and selectivity [70].

**Figure 11:**
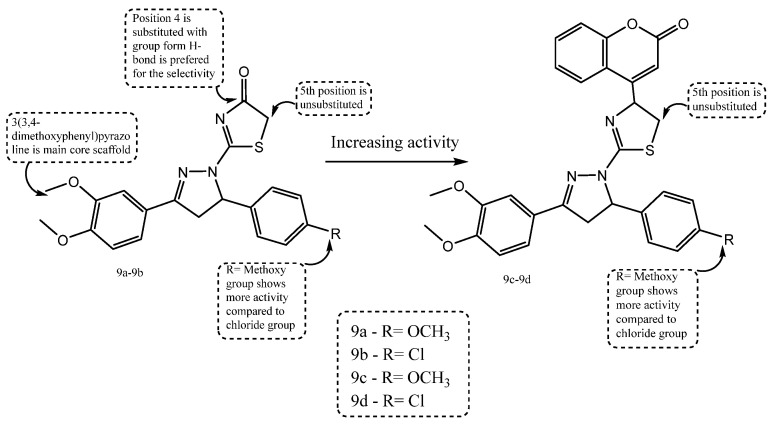
Structure of compounds **9a**–**9d** representing thiazolyl-pyrazoline derivatives.

A new series of thiazolyl–pyrazoline-based hybrid compounds, integrating thiazole and pyrazoline pharmacophores, was rationally designed and synthesized to explore dual epidermal growth factor receptor (EGFR) and human epidermal growth factor receptor 2 (HER2) inhibitory anticancer activity. The synthesized set of 18 derivatives was initially evaluated for in vitro anticancer activity against MCF-7 breast cancer cells, and several compounds showed strong cytotoxicity. Subsequent enzyme inhibition assays further assessed their ability to inhibit EGFR and HER2 kinases. Multiple derivatives demonstrated superior anticancer efficacy compared to the reference drug lapatinib, with structure–activity insights highlighting the effectiveness of the thiazolyl–pyrazoline framework in dual kinase inhibition. Among the evaluated series, compounds **9a**–**9d** were the most active, with IC_50_ values ranging from 3.37 to 5.64 µM against MCF-7 cells, all of which outperformed lapatinib (IC_50_ = 5.88 µM). As shown in Figure 11, compound **9c** demonstrated the lowest IC_50_ value against MCF-7 cells and the most potent dual inhibition of EGFR and HER2 kinases. Notably, compound **9c** exhibited EGFR IC_50_ = 0.005 µM and HER2 IC_50_ = 0.022 µM, confirming its promise as a dual-target anticancer agent [71].

**Figure 12:**
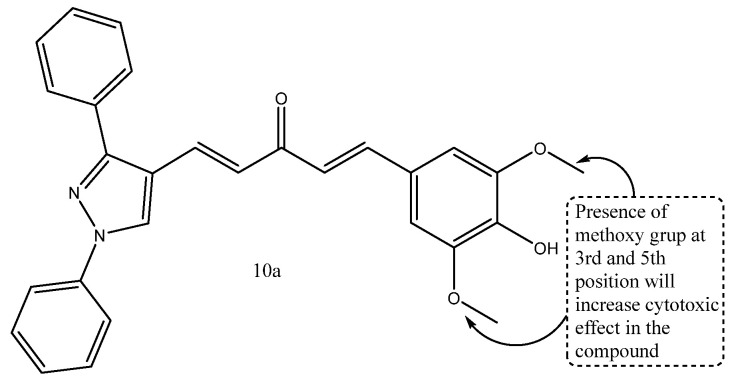
Structure of compound **10a** representing pyridazine-linked pyrazoline derivatives.

A series of curcumin-1,3-diphenyl-1*H*-pyrazole hybrid compounds was rationally designed and synthesized to explore their anticancer potential. The synthesized derivatives were evaluated for in vitro cytotoxicity against SW480 (colon), MDA-MB-231 (breast), and A549 (lung) cancer cell lines, and several compounds exhibited significant growth inhibition. Enzyme inhibition studies further assessed their effects on EGFR wild-type (EGFRWT) and mutant (EGFRL858R) kinases, while apoptosis assays were conducted to investigate the underlying mechanism of action. Among the evaluated series, compound **10a** emerged as the most potent analogue, displaying IC_50_ values of 4.86 μM (SW480), 4.89 μM (MDA-MB-231), and 4.44 μM (A549). As shown in Figure 12, compound **10a** exhibited consistent cytotoxic activity across the tested cancer cell lines and demonstrated moderate inhibitory activity against EGFR^WT (30%) and EGFR^L858R (41%). Furthermore, it induced apoptotic morphological changes and enhanced caspase-3 activity in SW480 cells, confirming its strong anticancer potential [72].

**Figure 13:**
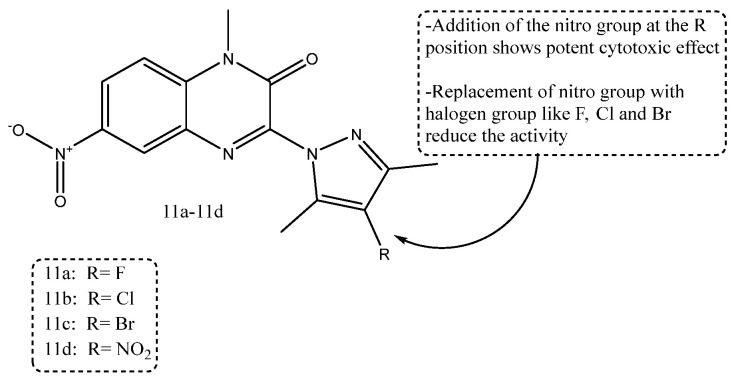
Structure of compounds **11a**–**11d** representing pyrazole-clubbed quinoxalinone derivatives.

A new series of quinoxalinone-based pyrazole derivatives was rationally designed and synthesized using a multitarget-directed drug design strategy to explore their anticancer potential. The synthesized compounds were evaluated for their in vitro antiproliferative activity against MCF-7, HCT-116, and A549 human cancer cell lines, where several derivatives demonstrated pronounced growth inhibition. In addition, their EGFR-inhibitory activity was assessed to identify a potential molecular target. Among the evaluated series, compounds **11a**–**11d** exhibited strong cytotoxic effects, with IC_50_ values of 3.91, 4.18, 5.07, and 2.04 μM (MCF-7), 4.13, 4.85, 5.96, and 2.69 μM (HCT-116), and 18.16, 16.92, 15.76, and 19.83 μM (A549), respectively. Notably, these compounds also showed potent EGFR inhibition, with IC_50_ values of 2.89, 3.71, 4.28, and 1.28 μM, all of which exceeded the activity of the reference inhibitor sorafenib (IC_50_ = 8.16 μM). Among them, compound **11d** emerged as the most potent analogue, demonstrating the lowest IC_50_ values across both cellular and EGFR inhibition assays. As shown in Figure 13, the structure–activity relationship (SAR) analysis indicates that the presence of a nitro group (–NO_2_) at the R position significantly enhances cytotoxic and EGFR inhibitory activity. In contrast, halogen substitution, such as F, Cl, and Br, reduces potency [73].

**Figure 14:**
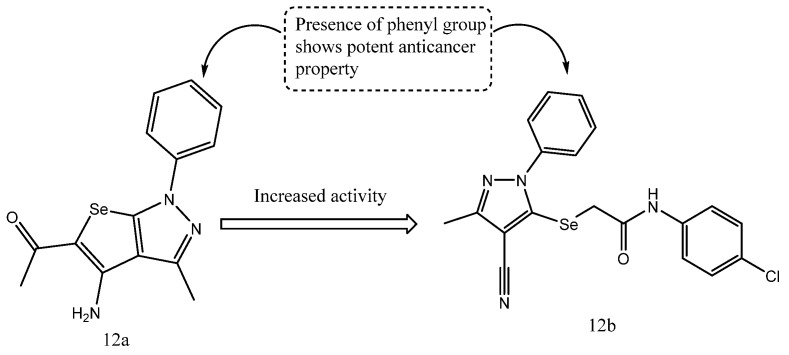
Structures of compounds **12a** and **12b** representing Se-alkylated pyrazole derivatives.

Se-alkylated pyrazole derivatives were prepared as antitumor analogues through alkylation of the pyrazole nucleus and were subsequently evaluated against the HepG2 cell line. Within this series, compounds **12a** and **12b** showed the strongest cytotoxic activity, with IC_50_ values of 4.30 μM and 4.49 μM, respectively. Reverse transcription quantitative polymerase chain reaction (RT-qPCR)-based analysis further demonstrated marked down-regulation of S100A4 and MMP-9, suggesting a possible effect on tumor progression. As shown in Figure 14, incorporation of a phenyl moiety appeared to enhance the biological activity of the pyrazolo [3,4-*d*]pyrimidine derivatives, in line with the observed suppression of metastasis-associated genes. Overall, compound **12a** was the most active member of the series and produced the strongest gene-downregulatory effect [74].

**Figure 15:**
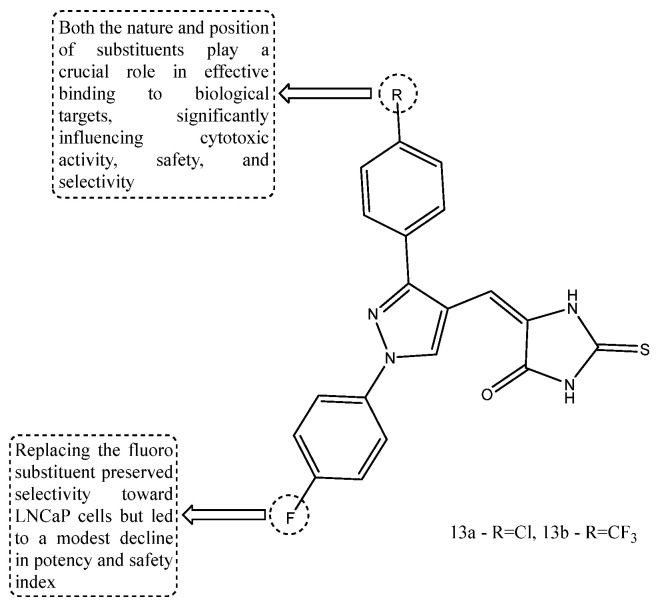
Chemical structure of compounds **13a** and **13b** representing pyrazolylmethylene-2-thioxoimidazolidin-4-one derivatives.

Pyrazolylmethylene-2-thioxo-imidazolidin-4-one derivatives were designed for the evaluation of anticancer activity in prostate cancer using a pharmacophore-based molecular hybridization strategy. The compounds were screened in androgen receptor (AR)-positive LNCaP cells, AR-negative PC-3 cells, and normal WI-38 cells. Compounds **13a** and **13b** showed the most notable antiproliferative effects, accompanied by apoptosis induction, increased caspase-3 expression, cell-cycle arrest, and inhibition of LNCaP cell proliferation. As illustrated in Figure 15, structure–activity relationship (SAR) analysis indicated that both the presence and the position of electron-withdrawing substituents on the benzene ring strongly influenced cytotoxic potency and selectivity. In particular, compound **13b**, bearing fluoro and trifluoromethyl groups, showed the best activity (IC_50_ = 5.22 ± 0.12 µM), together with high AR selectivity and a favorable safety profile [75].

Taken together, these studies support the pyrazole scaffold as a versatile platform for anticancer design; however, the reported SAR trends should be interpreted with caution. Direct cross-study comparison is limited by differences in cancer cell panels, assay formats, exposure times, reference compounds, and reporting metrics. In addition, some SAR conclusions appear to be series-specific rather than universally transferable across the broader pyrazole chemical space, particularly when comparing hybrid scaffolds and different substitution patterns. Thus, while recurrent motifs such as electron-withdrawing substituents, lipophilic aryl groups, and appropriately positioned heterocyclic linkers often correlate with improved activity, their contribution should be interpreted within the context of each individual chemotype. For ease of comparison, representative pyrazole series, biological models, and recurrent SAR observations discussed in this section are summarized in Table 1.

## 5. Mechanism of Action of Pyrazoles as Anticancer Agents

### 5.1. Tubulin Polymerization Inhibition

Microtubules are filamentous polymeric structures within the cytoplasm, consisting of α-tubulin and β-tubulin subunits. They are implicated in carcinogenesis and play a pivotal role in regulating numerous cellular functions, such as cell polarity, morphology, mitosis, intracellular transport, signal transduction, gene expression, and structural integrity [76,77]. Hence, it is now recognized as a vital target in the search for new anticancer agents. Tumor cell proliferation relies heavily on the uninterrupted polymerization and depolymerization of tubulin. Consequently, tubulin polymerization inhibitors have been developed as antimitotic agents and represent a fundamental class of chemotherapeutic compounds due to their ability to disrupt microtubule dynamics and inhibit cancer cell proliferation. Colchicine, the earliest known microtubule-destabilizing agent, inhibits mitosis [78,79].

**Figure 16:**
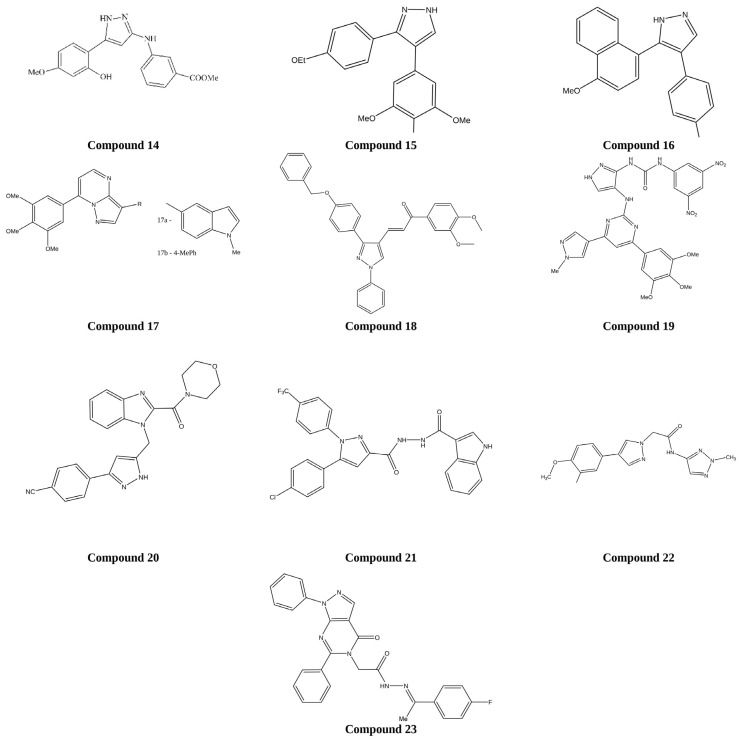
Chemical structures of compounds **14**–**23** representing pyrazole derivatives acting as tubulin polymerization inhibitors.

1*H*-benzofuro[3,2-*c*]pyrazole derivatives were designed and synthesized as potential tubulin polymerization inhibitors using a fused heterocyclic strategy. The series was evaluated for in vitro anticancer activity against K562, A549, and MCF-7 cell lines, with ABT-751 used as the reference standard. Compound 14 showed the strongest activity in the series and also inhibited tubulin polymerization with an IC_50_ value of 7.30 µM. As shown in Figure 16, its structural features appear to favor productive interaction with the tubulin binding site, thereby disrupting microtubule assembly and suppressing cancer cell proliferation [80].

A series of 3,4-diaryl pyrazole derivatives inspired by the structure of the tubulin inhibitor Combretastatin A 4 were synthesized using a rational design strategy aimed at targeting the colchicine-binding site of tubulin. Biological evaluation across six human cancer cell lines demonstrated remarkable antiproliferative activity for several analogues. Among the tested compounds, compound **15** emerged as the most potent, exhibiting IC_50_ values in the ultra-low nanomolar range (0.06–0.25 nM). In vivo studies further confirmed its efficacy, with a 5 mg/kg dose significantly reducing tumor size in a mouse breast cancer model. Mechanistic investigations revealed that compound **15** strongly inhibited tubulin polymerization (IC_50_ = 0.35 µM) and effectively blocked the colchicine-binding site, producing 96% inhibition at 5 µM and 90% inhibition at 0.5 µM [81].

Pyrazole–naphthalene derivatives were designed and synthesized via molecular hybridization to enhance their anticancer potential by inhibiting tubulin polymerization. The synthesized compounds were evaluated for cytotoxicity against the MCF-7 breast cancer cell line. Among the tested molecules, compound **16** exhibited significantly stronger cytotoxicity than cisplatin, with an IC_50_ value of 2.78 ± 0.24 µM. Additionally, compound **5** demonstrated tubulin polymerization-inhibitory activity nearly comparable to that of colchicine, with IC_50_ values of 4.6 µM and 6.7 µM, respectively. Overall, compound **16** was identified as the most potent anticancer agent in the series. As shown in Figure 16, the comparative analysis highlights the enhanced cytotoxicity of compound **16** relative to the reference drug cisplatin, and the notable tubulin inhibition exhibited by compound **5** compared to colchicine [82].

Pyrazolo[1,5-*a*]pyrimidine derivatives were designed and synthesized as potential tubulin polymerization inhibitors using an appropriate heterocyclic modification strategy. The synthesized compounds were evaluated for antiproliferative activity using the Cell Counting Kit-8 (CCK-8) assay in vitro against five cancer cell lines (HeLa, MCF-7, A549, HCT-116, and B16F10), with colchicine and paclitaxel as reference compounds. Among the tested molecules, compounds **17a** and **17b** exhibited the most potent cytotoxic activity across the evaluated cell lines, with IC_50_ values ranging from 0.003 µM to 0.048 µM, and were identified as the most active derivatives in the series. The representative pyrazolo[1,5-*a*]pyrimidine scaffold shown as compound **17** in Figure 16 reflects the core structural motif of this highly active series and highlights the features associated with the enhanced anticancer potential of compounds **17a** and **17b** [83].

(E)-3-(3-(4-(benzyloxy)phenyl)-1-phenyl-1*H*-pyrazol-4-yl)-1-phenylprop-2-en-1-one derivatives were designed and synthesized using an appropriate chalcone-based hybridization strategy to evaluate their anticancer potential via tubulin polymerization inhibition. The synthesized compounds were assessed for cytotoxicity against breast (MCF-7), cervical (SiHa), and prostate (PC-3) cancer cell lines, as well as against normal human embryonic kidney (HEK) cells. Among the tested molecules, compound **18** exhibited the most potent antiproliferative activity, with IC_50_ values of 2.13 ± 0.80 µM against MCF-7, 4.34 ± 0.98 µM against SiHa, and 4.46 ± 0.53 µM against PC-3 cancer cell lines, while showing no significant toxicity toward normal HEK cells. These findings indicate its selective cytotoxicity toward cancerous cells and identify it as the most active derivative in the series. As shown in Figure 16, the structural framework of compound **18** contributes to its enhanced anticancer efficacy and improved safety profile compared to other evaluated analogues [84].

Pyrimidine–pyrazole-substituted aryl urea derivatives were synthesized as potential antiproliferative agents using a rational hybridization strategy that combines pyrimidine and pyrazole pharmacophores. The synthesized compounds were evaluated for anticancer activity against human cancer cell lines MCF-7, A549, COLO205, and A2780. Several derivatives exhibited significant cytotoxic activity, indicating the effectiveness of this scaffold for anticancer drug development. As shown in Figure 16 among the tested compounds, compound **19** emerged as the most potent analogue, outperforming the reference drug etoposide across all evaluated cell lines with IC_50_ values ranging from 0.01 to 0.65 µM. Furthermore, molecular docking studies indicated that the active derivative acts as a tubulin inhibitor, occupying the colchicine binding site and supporting its potential anticancer mechanism of action [85].

Morpholine–benzimidazole–pyrazole hybrid derivatives were designed and synthesized via molecular hybridization to evaluate their potential as tubulin polymerization inhibitors for cancer therapy. The synthesized compounds were evaluated for cytotoxicity against MCF-7, PC-3, and A549 cancer cell lines. Among the tested molecules, compound **20** exhibited significantly greater cytotoxicity than the standard drug combretastatin A-4, with IC_50_ values of 0.042 µM (MCF-7), 0.61 µM (PC-3), and 0.76 µM (A549), along with potent inhibition of tubulin polymerization (IC_50_ = 0.35 µM). Molecular docking studies further confirmed its strong binding interaction at the α,β-tubulin interface, and compound **20** was identified as the most promising lead molecule in the series for the development of next-generation tubulin-targeting anticancer agents. As shown in Figure 16, the structural configuration of compound **20** contributes to its enhanced binding affinity and superior biological activity compared to the reference compound [86].

Pyrazole–carbohydrazide derivatives incorporating an indole moiety were designed and synthesized using a combretastatin A-4 (CA-4)-inspired molecular hybridization strategy to evaluate their potential to induce mitotic catastrophe via tubulin polymerization inhibition. The synthesized compounds were evaluated for antiproliferative activity against HeLa, HepG2, A549, and MCF-7 cancer cell lines. Among the tested molecules, compound **21** exhibited notable tubulin polymerization inhibitory activity (IC_50_ = 2.23 µM) along with promising antiproliferative effects, with GI_50_ values of 6.23 µM (HeLa), 0.71 µM (HepG2), 0.24 µM (A549), and 0.92 µM (MCF-7), and was identified as the most potent derivative in the series. As shown in Figure 16, the structural features of compound **21** enhance its interaction with the tubulin binding site, resulting in effective inhibition of microtubule assembly and cancer cell proliferation [87].

Pyrazole derivatives were designed and synthesized to evaluate their anticancer potential using a targeted molecular modification strategy. The synthesized compounds were biologically assessed for their mechanism of action using differential nuclear staining, whole ribonucleic acid (RNA) sequencing, Connectivity Map (CMap) analysis, and biochemical assays. Among the tested molecules, compound **22** exhibited potent anticancer activity with low cytotoxicity toward non-cancerous cells. It effectively induced apoptosis, triggered cell cycle arrest in the S and G2/M phases, and modulated gene expression in a manner comparable to established tubulin inhibitors. These findings highlight its selective cytotoxic potential and favorable safety profile. As shown in Figure 16, the structural features of compound **22** contribute to its biological behavior, supporting its identification as the most promising candidate in the series for future clinical development as a tubulin-targeting anticancer agent [88].

Pyrazolo[3,4-*d*]pyrimidine derivatives were designed by incorporating key pharmacophoric features of known EGFR inhibitors in order to enhance targeted anticancer activity. After evaluation against MCF-7 breast cancer cells, compound **23** emerged as the leading analogue, showing an IC_50_ value of 2.89 µM, close to that of toceranib and lower than that of doxorubicin. Structure–activity relationship (SAR) analysis indicated that the introduction of aryl or heteroaryl groups through a hydrophilic linker improved anticancer potency. Molecular docking also supported strong binding of compound **23** within the EGFR kinase domain, consistent with its profile as the most promising selective EGFR-oriented derivative in the series [52].

### 5.2. Kinase Inhibition

**Figure 17:**
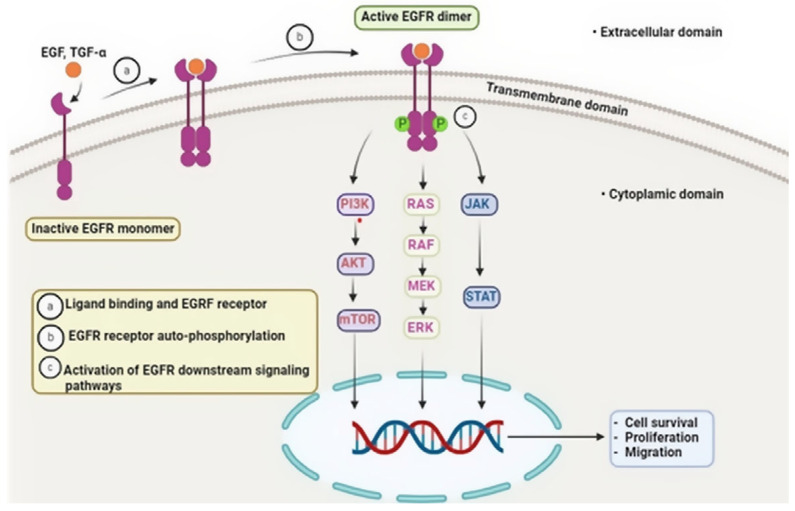
Schematic representation of EGFR activation and downstream signaling pathways.

Protein kinases are crucial therapeutic targets for the development of anticancer drugs because they regulate cell proliferation, differentiation, and apoptosis. One promising method of treating cancer is the creation of tiny compounds that block these kinases. A recent investigation reported the identification of 42 protein kinase inhibitors bearing a unique, unfused pyrazole moiety, all of which are undergoing clinical evaluation. The molecules demonstrated targeted inhibition across multiple oncogenic kinases, including protein kinase B (AKT), cyclin-dependent kinases (CDKs), epidermal growth factor receptor/vascular endothelial growth factor receptor (EGFR/VEGFR), mitogen-activated protein kinase (MAPK), B-Raf proto-oncogene serine/threonine kinase (BRAF), Janus kinase (JAK), hepatocyte growth factor receptor (c-Met), and Bruton tyrosine kinase (BTK), highlighting their broad-spectrum anticancer potential. Clinically relevant lead compounds such as afuresertib (AKT inhibitor), lazertinib (EGFR inhibitor), dexmetinib (MAPK pathway inhibitor), encorafenib (BRAF inhibitor), ruxolitinib (JAK inhibitor), crizotinib (c-Met inhibitor), and pirobrutinib (BTK inhibitor) have shown considerable therapeutic efficacy and continue to hold promise for future drug development. Figure 17 provides a mechanistic overview of the EGFR-centered signaling network relevant to this section, showing how receptor activation propagates through the phosphoinositide 3-kinase/protein kinase B/mammalian target of rapamycin (PI3K/AKT/mTOR), rat sarcoma/rapidly accelerated fibrosarcoma/mitogen-activated protein kinase kinase/extracellular signal-regulated kinase (RAS/RAF/MEK/ERK), and Janus kinase/signal transducer and activator of transcription (JAK/STAT) axes that govern cell survival, proliferation, and migration—processes frequently modulated by the pyrazole-based kinase inhibitors discussed below [44,48,89].

**Figure 18:**
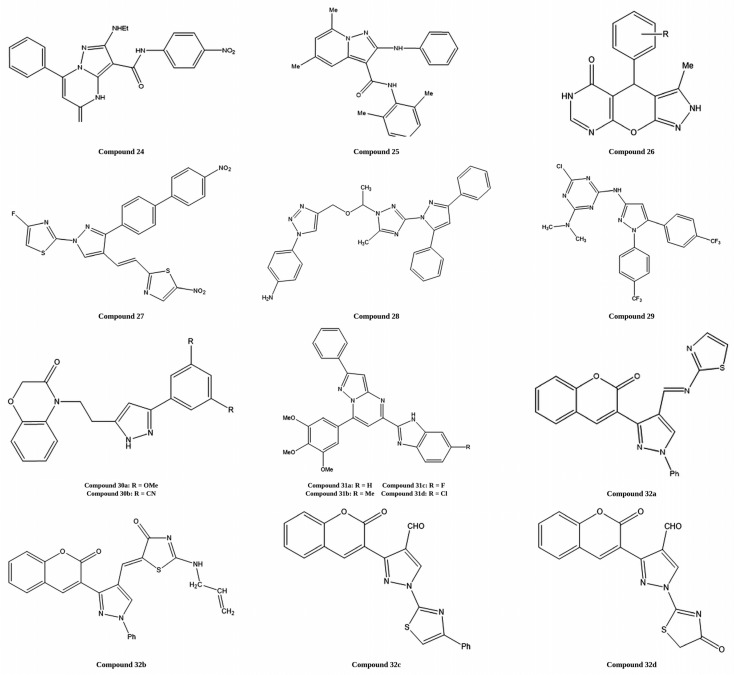
Chemical structure of compounds **24**–**32** representing pyrazole derivatives as kinase inhibitors.

Pyrazolopyrimidine derivatives were synthesized and evaluated for in vitro antiproliferative activity against HCT-116 and MCF-7 cancer cell lines using the MTT assay. Compounds 24 and 25 showed the best activity within the series, with compound **24** being the most potent and displaying an IC_50_ value of 1.51 µM against HCT-116 cells. As indicated in Figure 18, the observed activity suggests that structural modification of this scaffold can meaningfully improve antiproliferative effects in colorectal cancer models [90].

Pyrazolo[4′,3′:5,6]pyrano[2,3-*d*]pyrimidin-5(2*H*)-one derivatives were designed and synthesized using a fused heterocyclic framework to evaluate their anticancer efficacy. The synthesized compounds were evaluated for cytotoxicity against MCF-7, Caco-2, HeLa, and HepG2 cancer cell lines. Among the tested molecules, compounds **26a** and **26b** exhibited potent cytotoxicity, with IC_50_ values of 13–17 µg/mL, closely matching the activity of the reference drug doxorubicin. Among them, compound **26a** was identified as the most active derivative in the series. As shown in Figure 18, structural variations between **26a** and **26b** contribute to their comparable yet slightly differing cytotoxic profiles [91].

Thiazole–pyrazole derivatives were designed and synthesized via molecular hybridization to evaluate their in vitro anticancer potential. The synthesized compounds were assessed for cytotoxicity against the MCF-7 and HeLa cancer cell lines. Among the tested molecules, compound **27** exhibited high cytotoxicity, with an IC_50_ value of 30.7 µg/mL against MCF-7 cells and below 10 µg/mL against HeLa cells, demonstrating activity against HeLa cells comparable to the standard chemotherapy drug adriamycin. Based on its superior antiproliferative profile, compound **27** was identified as the most potent derivative in the series. As shown in Figure 18, the structural features of compound **27** may account for its enhanced cytotoxic performance across different cancer cell lines [92,93].

1,2,3-Triazole–pyrazole hybrid derivatives were designed and synthesized via Cu-catalyzed azide–alkyne cycloaddition (CuAAC) click chemistry to enhance anticancer activity by inhibiting EGFR. The synthesized compounds were evaluated for their in vitro cytotoxicity against the HepG2, HCT-116, and MCF-7 cancer cell lines. Among the tested molecules, compound **28** exhibited the most potent anticancer activity, with IC_50_ values of 12.22, 14.16, and 14.64 µM, respectively, comparable to those of doxorubicin. Molecular docking studies further confirmed its strong binding affinity toward the EGFR kinase domain, identifying compound **28** as the most active derivative in the series. As illustrated in Figure 18, the structural features responsible for its enhanced receptor binding and anticancer activity are clearly presented [94].

1,3,5-Triazine-based pyrazole hybrid derivatives were designed and synthesized using a molecular hybridization strategy to target EGFR tyrosine kinase. The synthesized compounds were biologically evaluated for EGFR inhibitory activity and anticancer potential across multiple human cancer cell lines, including MCF-7, HepG2, HCT-116, PC-3, LoVo, and drug-resistant LoVo/DX cells. The derivatives exhibited IC_50_ values ranging from 229.4 to 596.2 nM, comparable to standard drugs such as doxorubicin and erlotinib. Structure–activity relationship analysis revealed that the nature and position of substituents on the phenyl ring, particularly electron-withdrawing groups at the *para* position, significantly enhanced activity. Among the series, compound **29**, bearing a *para*-trifluoromethyl substituent, demonstrated the highest EGFR inhibition with an IC_50_ value of 229.4 nM and was identified as the most potent derivative. As shown in Figure 18, the presence of the *para*-trifluoromethyl group significantly enhances the interaction of compound **29** within the EGFR binding pocket, thereby improving its inhibitory activity [95].

3,5-Disubstituted 1,4-benzoxazine–pyrazole hybrid derivatives were designed and synthesized via molecular hybridization to evaluate their antiproliferative activity via EGFR inhibition. The synthesized compounds were evaluated for their in vitro anticancer activity against the MCF-7, A549, HeLa, and PC-3 human cancer cell lines. Among the tested molecules, compounds **30a** and **30b** exhibited significant cytotoxicity, with IC_50_ values ranging from 2.82 to 6.28 µM, comparable to those of the standard drug etoposide. Further EGFR inhibition studies revealed superior potency, with IC_50_ values of 0.6124 µM for compound **30a** and 0.5132 µM for compound **30b.** Molecular docking analysis also confirmed their strong interaction within the EGFR active site, identifying compound **30b** as the most potent derivative in the series. As shown in Figure 18, the structural configuration of these derivatives contributes to their enhanced EGFR inhibitory activity and anticancer potential [96].

Benzimidazole-linked pyrazolo[1,5-*a*]pyrimidine derivatives were designed and synthesized via molecular hybridization to evaluate their antiproliferative activity via EGFR inhibition. The synthesized compounds were assessed for their in vitro anticancer potential against MCF-7, A549, HeLa, and SiHa cancer cell lines, as well as for their toxicity in normal MRC-5 cells. Among the tested molecules, compounds **31a**–**31d** exhibited potent selective cytotoxicity with micro- to nanomolar IC_50_ values and low toxicity toward normal cells. Mechanistic investigations revealed that these derivatives induced apoptosis by downregulating EGFR, p-EGFR, STAT3, p-STAT3, Bcl-2, and procaspase-9, while upregulating pro-apoptotic proteins such as p53, p21, and BAX. EGFR inhibition studies demonstrated IC_50_ values of 0.82, 0.31, 0.37, and 0.29 µM for compounds **31a**–**31d**, respectively, surpassing the standard drug erlotinib. Based on these findings, compound **31c** was identified as the most potent derivative in the series. As shown in Figure 18, the structural features of these derivatives contribute to their enhanced EGFR inhibitory activity and apoptosis-inducing potential [97].

Coumarin–pyrazole–thiazole hybrid derivatives were synthesized through condensation of thiazole intermediates and then evaluated against MCF-7, HepG2, and HCT-116 tumor cell lines. Several compounds showed marked cytotoxicity and were associated with cancer cell death and cell-cycle arrest. In this group, compounds **32a**–**32d** displayed activity comparable to doxorubicin and also showed strong binding affinity toward cyclin-dependent kinase 8 (CDK-8) in docking studies, supporting their potential as promising anticancer leads. As illustrated in Figure 18, the overall hybrid framework appears to favor productive interaction with the CDK-8 binding domain [98].

### 5.3. Multitargeted Kinase Inhibition

Multitarget kinase inhibitors offer a promising approach to precision cancer therapy by simultaneously disrupting multiple tumor signaling pathways, thereby helping minimize the development of drug resistance. Sorafenib, a pioneering multi-kinase inhibitor, has set a precedent by targeting key receptors, including vascular endothelial growth factor receptor (VEGFR), platelet-derived growth factor receptor (PDGFR), and KIT proto-oncogene receptor tyrosine kinase (KIT), effectively blocking both angiogenesis and proliferation. Therefore, sorafenib has been widely adopted as the primary treatment option for advanced stages of hepatocellular and renal cell carcinomas [99].

**Figure 19:**
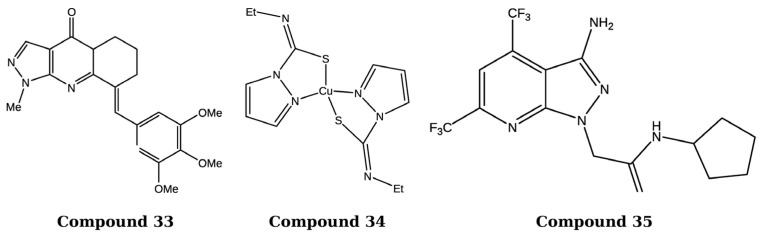
Chemical structure of compounds **33**–**35** representing pyrazole derivatives as Multitargeted kinase inhibitors.

Pyrazolo[3,4-*d*]pyrimidine derivatives bearing carbon-aryl (heteryl)idene moieties were synthesized and evaluated for their anticancer potential across 15 different human cancer cell lines. The synthesized compounds demonstrated notable antiproliferative activity, and further in vivo evaluation in HT-29 xenograft mouse models confirmed their cytotoxicity. Mechanistic studies revealed that the active derivatives induced G2/M phase cell cycle arrest by disrupting mitochondrial membrane potential, inhibited VEGFR-2 kinase via hydrogen-bonding interactions with Cys-919, and interfered with tubulin polymerization by binding to the colchicine-binding site. Among the series, compound **33** exhibited the most potent activity with IC_50_ values ranging from 0.03 to 6.561 µM, suggesting that its anticancer effect may be mediated through anti-angiogenic activity and microtubule disruption. As shown in Figure 19, the structural features of compound **33** contribute to its enhanced biological activity and potential mechanism of action involving inhibition of tumor vascularization and microtubule dynamics [100].

Pyrazole-based ligands incorporating bidentate nitrogen and sulfur donor atoms were synthesized and subsequently complexed with copper(II) ions to evaluate their antitumor potential against MCF-7 breast cancer cells. The resulting copper(II) complexes exhibited enhanced cytotoxicity compared to their uncoordinated ligands and demonstrated stronger binding interactions with EGFR and CDK2 in molecular docking studies. Among the synthesized derivatives, compound **34** showed the highest cytotoxicity against MCF-7 cells, with an IC_50_ of 20.70 µM, outperforming the other tested ligands in the series. As shown in Figure 19, the structural characteristics of compound **34** contribute to its enhanced antiproliferative activity against breast cancer cells [101].

Pyrazolo[3,4-*b*]pyridine derivatives incorporating amide and amino acid functionalities were synthesized and evaluated for their antiproliferative activity against A549, MCF-7, DU145, and HeLa cancer cell lines. The synthesized compounds demonstrated promising cytotoxicity compared with the standard drug 5-fluorouracil. Molecular docking studies further indicated strong binding interactions of the active derivatives with EGFR and HER2 targets. Among the series, compound **35** exhibited the highest cytotoxic potency, with IC_50_ values of 21.2, 18.4, 19.2, and 25.3 µM against the respective cancer cell lines, along with a binding energy of –10.98 kcal/mol and an inhibition constant of 23.91 µM. As shown in Figure 19, the structural framework of compound **35** supports its enhanced binding affinity and cytotoxic potential compared to other derivatives in the series [102].

### 5.4. Other Targets Inhibition

#### 5.4.1. DNA Binding Agents

A novel group of anticancer agents, DNA-binding inhibitors, functions by directly targeting DNA to halt cancer cell proliferation. These agents differ from traditional chemotherapy by offering selective action, innovative mechanisms, lower side effects, and the potential to overcome resistance—highlighting their potential in future cancer treatment strategies [103].

**Figure 20:**
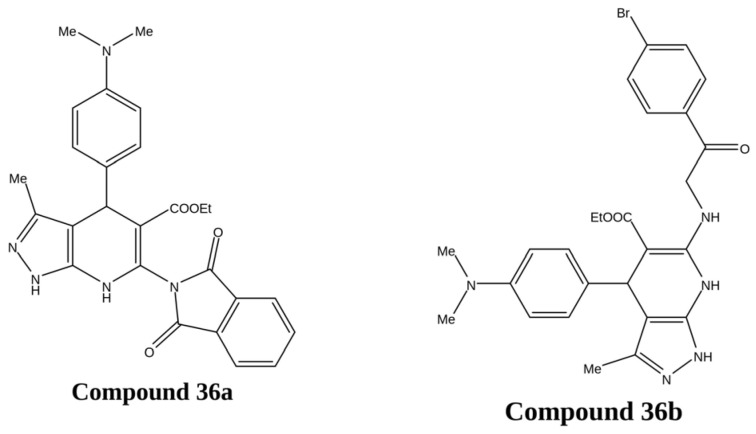
Chemical structure of compounds **36a** and **36b** representing pyrazole derivatives as DNA binding inhibitors.

A novel series of pyrazolo[3,4-*b*]pyridine derivatives was synthesized using a multistep synthetic strategy and evaluated for in vitro anticancer activity against HepG2, MCF-7, and HeLa cancer cell lines. The biological evaluation revealed that the synthesized compounds exhibited significant cytotoxicity, with reduced toxicity toward normal human amniotic-derived epithelial (WISH) and WI-38 cell lines. DNA-binding studies further demonstrated a strong interaction with DNA, indicating a mechanism comparable to that of the standard drug doxorubicin. Among the tested compounds, **36a** and **36b** emerged as the most active derivatives, showing marked cytotoxicity (IC_50_ values of 3.11–4.91 µM and 4.06–4.24 µM, respectively) and notable DNA-binding affinity (IC_50_ values of 27.13 µM and 29.15 µM, respectively). As shown in Figure 20, the structural framework of these derivatives contributes to their enhanced cytotoxic potential and improved interaction with DNA [104].

#### 5.4.2. Topoisomerase Inhibitors

DNA topoisomerases, especially type IIA, represent attractive targets for cancer therapy. These enzymes play a crucial role in maintaining DNA’s topological stability by relieving excessive winding or unwinding during replication. Research indicates that type IIA topoisomerases are often overexpressed and hyperactive in cancer cells compared to normal cells. Thus, specifically targeting and suppressing the activity of these enzymes in cancer cells may effectively destroy cancer cells while sparing healthy ones [105].

A series of spiro[indenoquinoxalinepyrrolizidine]-pyrazole conjugates was synthesized and evaluated for in vitro anticancer activity against HeLa cells. Biological screening showed stronger inhibition of cancer cell proliferation than that produced by camptothecin. Within this series, compound **37** displayed the highest activity, with an IC_50_ value of 1.93 ± 0.18 µM [106].

**Figure 21:**
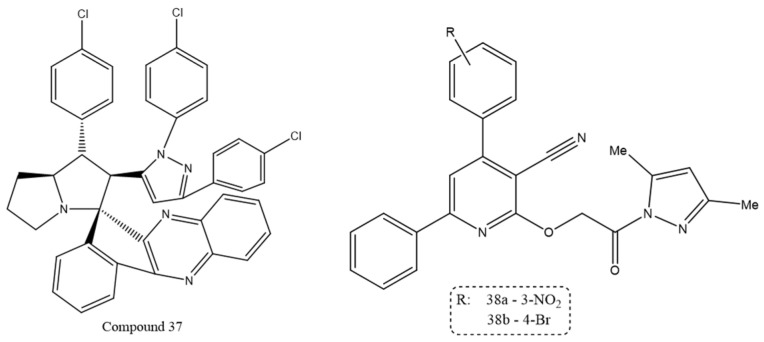
Chemical structure of compounds **37**, **38a** and **38b** representing pyrazole derivatives as Topoisomerase inhibitors.

Another series of pyrazole derivatives was developed and evaluated for cytotoxicity against Caco-2 and MCF-7 cancer cell lines. The compounds showed notable antiproliferative effects, and molecular docking suggested strong binding to the topoisomerase-IIβ active site. Compounds **38a** and **38b** were the most active analogues, with pronounced activity against Caco-2 cells and log IC_50_ values of –0.657 and –0.498 µM at 0.1 and 1 µM, respectively, exceeding the activity of docetaxel. As shown in Figure 21, their structural features may underlie the enhanced cytotoxic profile observed in this series [107].

#### 5.4.3. HDAC Inhibitors

Targeting histone deacetylases (HDACs) represents a validated and promising avenue in cancer treatment. Several HDAC inhibitors have successfully progressed to clinical use, with six: vorinostat, romidepsin, belinostat, panobinostat, chidamide, and pracinostat having received U.S. Food and Drug Administration (FDA) approval. These drugs have shown effectiveness in treating T-cell lymphomas and recurrent multiple myeloma. However, their use in solid tumors remains limited due to side effects such as thrombosis, leukopenia, and anemia. Dysregulated HDAC activity is associated with various cancers, supporting the rationale for developing HDAC inhibitors [108,109].

**Figure 22:**
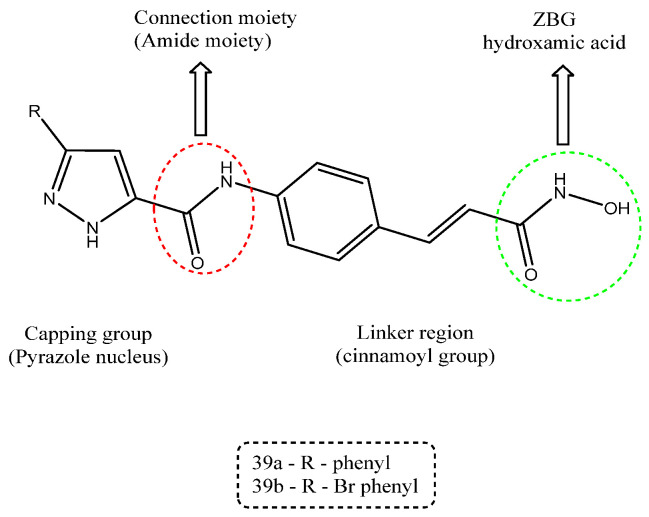
Chemical structure of compounds **39a** and **39b** representing pyrazole derivatives as HDAC inhibitors.

A series of hydroxamic acid derivatives incorporating a pyrazole cap and a cinnamoyl linker was synthesized using an appropriate synthetic strategy and evaluated for histone deacetylase (HDAC) inhibitory activity. The biological studies demonstrated that the synthesized compounds exhibited potent HDAC inhibition and effectively reduced cancer cell proliferation in a dose- and time-dependent manner. Molecular docking studies further confirmed their effective binding within the histone deacetylase 8 (HDAC8) hydrophobic pocket, along with zinc ion coordination by the hydroxamic acid moiety. Among the tested derivatives, compounds **39a** and **39b** emerged as the most active analogues, displaying strong HDAC inhibitory activity (IC_50_ values ranging from 1.3 to 6.3 µM) and antiproliferative activity (IC_50_ values of 5.34 and 5.61 µM, respectively). Structure–activity relationship analysis highlighted the essential role of the hydroxamic acid group in zinc chelation, while the cinnamoyl linker and pyrazole moiety contributed to enhanced isoform selectivity and potency. As illustrated in Figure 22, the pharmacophoric features, including the capping pyrazole nucleus, the cinnamoyl linker region, and the hydroxamic acid zinc-binding group (ZBG), are clearly depicted, which explains their improved HDAC inhibitory activity [110].

Collectively, the mechanistic data indicate that pyrazole derivatives can engage a broad spectrum of cancer-relevant targets, including tubulin, EGFR-related signaling, topoisomerases, DNA, and HDACs. Nevertheless, the depth of mechanistic validation is not uniform across the literature. For several series, target assignment is supported by enzyme inhibition, apoptosis, or cell-cycle assays, and, in selected cases, in vivo studies; however, in many reports, the proposed mechanism relies mainly on cytotoxicity data supplemented by molecular docking. This limits the strength of causal interpretation, because docking alone cannot confirm target engagement, pathway selectivity, or the relative contribution of off-target effects. Future studies would therefore benefit from more rigorous and standardized mechanistic validation, including orthogonal biochemical assays, target-engagement studies, selectivity profiling, and better integration of in vitro and in vivo evidence. The main molecular targets, representative compounds, and the level of supporting biological evidence discussed in this section are summarized in Table 2.

## 6. Future Perspectives

Pyrazoles, as nitrogen-containing aromatic scaffolds, remain highly attractive in anticancer drug discovery because of their favorable chemical properties and broad biological potential. Current research points to several particularly promising directions, including target-focused design, combination strategies, and nanotechnology-based delivery. Further progress will depend on continued lead optimization, exploration of additional molecular targets, and the identification of biomarkers that may support more precise therapeutic application. Future work is also likely to focus on clarifying the molecular basis of pyrazole-mediated anticancer effects, including their influence on signaling pathways, epigenetic regulation, cancer stem cells, and resistance mechanisms. Equally important will be the development of rational combination approaches and the design of derivatives with improved pharmacokinetic and pharmacodynamic profiles.

From a translational perspective, one of the main challenges in this field is the gap between promising early-stage activity and the level of evidence required for drug development. Many pyrazole-based candidates are still supported mainly by single-study in vitro data, whereas comparative pharmacokinetic, toxicological, selectivity, and in vivo efficacy data remain limited. More standardized biological evaluation and reporting would improve cross-study comparability and support more reliable prioritization of leads for further development. Overall, pyrazole derivatives remain a promising class of anticancer candidates, but their successful translation will require not only continued scaffold optimization, but also stronger biological validation and a more consistent evidence base.

An additional translational aspect that merits attention is that a subset of pyrazole-based anticancer candidates has already progressed beyond single-study in vitro screening to broader preclinical evaluation, including selected in vivo efficacy studies and mechanism-oriented follow-up in animal models [111,112]. This translational space also extends to natural-product-inspired hybrid design. In particular, steroidal and secosteroidal pyrazoline/pyrazole derivatives have emerged as promising scaffolds with anticancer potential, while pyrazole-containing terpenoid hybrids, especially pentacyclic triterpenoid systems, represent another relevant direction for further optimization [112,113,114]. In parallel, the patent literature indicates continued medicinal chemistry interest in pyrazole-containing anticancer agents, including programs directed toward CDK2, CDK12/13, KRAS-mutant cancers, and ErbB/HER2-related signaling [115,116,117,118]. Together, these observations support the view that the pyrazole scaffold is not only synthetically versatile but also increasingly relevant from a broader preclinical and translational perspective.

## 7. Materials and Methods

This narrative review was based on a targeted literature search of PubMed, Scopus, and Google Scholar updated up to March 2026, with emphasis on studies published between 2000 and 2026. Priority was given to recent original research articles describing the synthesis, biological evaluation, molecular targets, and structure–activity relationships of pyrazole derivatives with anticancer activity, while older seminal references were included selectively when necessary for historical, chemical, or mechanistic context. Selected recent review articles, including studies published in 2025–2026, were also incorporated to provide updated background and contextual support. The reference lists of relevant articles were additionally screened to identify further studies for inclusion.

## 8. Conclusions

Heterocyclic compounds, especially nitrogen-containing scaffolds such as pyrazoles, continue to attract considerable scientific interest because of their structural diversity and broad pharmacological potential. In the anticancer field, pyrazole derivatives have shown promising activity through multiple mechanisms, including inhibition of tubulin polymerization, kinase modulation, and induction of apoptosis, which underscores the versatility of this scaffold for medicinal chemistry optimization. Structure–activity relationship studies have further demonstrated that appropriate substitution patterns can substantially influence potency, selectivity, and target engagement. At the same time, the literature reviewed here indicates that many currently available findings remain difficult to compare directly because of differences in biological models, assay conditions, reference compounds, and reporting formats. Moreover, although several pyrazole-based candidates have shown encouraging activity, most remain at an early preclinical stage and are still supported predominantly by in vitro data, whereas comparative in vivo, toxicological, pharmacokinetic, and selectivity studies are often limited. For this reason, further progress in the field will depend not only on continued scaffold optimization but also on more rigorous mechanistic validation and more standardized biological evaluation. Overall, the pyrazole core remains a highly valuable platform in anticancer drug discovery, and this review aims to provide a useful framework for the rational development of next-generation pyrazole-based anticancer agents.

## Figures and Tables

**Table 1:** Representative pyrazole-based anticancer series and recurrent SAR observations discussed in Section 4.

Scaffold/Series	Representative Compound(s)	Main Model(s)	Best Reported Activity	Key SAR Takeaway
1,2,3-Triazole-linked 3-(1,3-diphenyl-1*H*-pyrazol-4-yl)acrylates	Compound **1** [63]	MCF-7, A549, HCT-116, HT-29	IC50 1.764–4.496 μM	Electron-donating groups on the diphenyl pyrazole core improved activity, whereas an electron-withdrawing nitro-substituted phenyl ring on the triazole side favored stronger target interactions.
Pyrazole-1,2,4-triazole hybrids	Compounds **3a**, **3b** [65]	A-549, MCF-7, HCT-116, PC-3	IC50 3.66–6.48 μM	A C-4 triazole linker, a C-3 methyl isostere, an oxime NO-donor moiety, and a sulfamoyl group were associated with improved potency and selectivity.
N-Aryl acetamide pyrazoles	Compounds **4a**, **4b** [66]	MCF-7	IC50 0.604–0.665 μM	Methoxy-rich aryl substitution enhanced activity, while the amide linkage contributed to favorable polarity and reduced aggregation.
4-Aminopyrazoles	Compound **5b** [67]	HeLa	IC50 0.074 μM	Substituent changes at N1 and C5 strongly affected potency; NH2/NO-related substitution patterns and methyl groups were particularly favorable.
Pyrazole-pyrazoline hybrids	Compound **8** [70]	A549, SiHa, COLO205, HepG2	IC50 2.09–4.94 μM; HaCaT > 50 μM	An unsubstituted ring B and methoxy substitution on ring C improved activity, while increasing electronegativity reduced efficacy.
Thiazolyl-pyrazoline dual kinase hybrids	Compound **9c** [71]	MCF-7; EGFR/HER2	EGFR IC50 0.005 μM; HER2 IC50 0.022 μM	The thiazolyl-pyrazoline framework enabled strong dual EGFR/HER2 inhibition and supported apoptosis-related effects.
Quinoxalinone-pyrazole derivatives	Compound **11d** [73]	MCF-7, HCT-116, A549; EGFR	Cell IC50 2.04–19.83 μM; EGFR IC50 1.28 μM	A nitro substituent at the R position was more favorable than halogens for both cytotoxic and EGFR-inhibitory activity.
Pyrazolylmethylene-2-thioxoimidazolidin-4-one derivatives	Compound **13b** [75]	LNCaP, PC-3, WI-38	IC50 5.22 +/− 0.12 μM	Electron-withdrawing fluoro and trifluoromethyl substituents improved potency, androgen-receptor selectivity, and the safety profile.

**Table 2:** Representative molecular targets, illustrative pyrazole derivatives, and the level of mechanistic support discussed in Section 5.

Target/Mechanism	Representative Compounds	Evidence Supporting Assignment	Representative Activity	Interpretative Takeaway
Tubulin polymerization inhibition	Compounds **15**, **20**, **21**, **22** [81,86,87,88]	Tubulin assays, cell-cycle/apoptosis studies, transcriptomic profiling, and selected in vivo evidence	Compound **15**: 0.06–0.25 nM in cells; tubulin IC50 0.35 μM. Compound **20**: tubulin IC50 0.35 μM.	Tubulin is among the best-supported targets in this review, although the depth of validation still varies between series.
EGFR/HER2-directed kinase inhibition	Compounds **9c**, **23**, **28**–**31** [52,71,94,95,96,97]	Enzyme inhibition, docking, apoptosis/protein-expression assays, and selective normal-cell comparisons in some series	Compound **9c**: EGFR IC50 0.005 μM; HER2 IC50 0.022 μM. Compounds **31a**–**31d**: EGFR IC50 0.29–0.82 μM.	EGFR-centered design is a recurring theme, but the strength of mechanistic confirmation is uneven across studies.
Other kinase pathways/multitarget kinase inhibition	Compounds **24**–**27**, **32**–**35** [90,91,92,93,98,100,101,102]	Cytotoxicity screening, docking, kinase-related assays, and selected in vivo validation	Compound **33**: IC50 0.03–6.561 μM across 15 cell lines with VEGFR-2/tubulin-related effects.	Multitarget design may help address resistance, but it also makes it harder to assign a single dominant mechanism.
DNA binding	Compounds **36**, **36b** [104]	DNA-binding studies plus cytotoxicity evaluation against cancer and normal cells	Cell IC50 3.11–4.91 μM and 4.06–4.24 μM; DNA-binding IC50 27.13 and 29.15 μM.	Direct DNA interaction is supported experimentally, although the broader selectivity profile still remains limited.
Topoisomerase inhibition	Compounds **37**, **38a**, **38b** [106,107]	Cytotoxicity assays and docking against topoisomerase-related targets	Compound 37: HeLa IC50 1.93 +/− 0.18 μM; compounds **38a**,**38b** active against Caco-2 cells.	The reported data are promising, but more direct biochemical confirmation of topoisomerase inhibition would strengthen causality.
HDAC inhibition	Compounds **39a**, **39b** [110]	HDAC inhibition assays, docking, and antiproliferative studies	HDAC IC50 1.3–6.3 μM; antiproliferative IC50 5.34 and 5.61 μM.	These findings support the pyrazole scaffold as a useful cap group in HDAC-oriented design, especially when paired with an appropriate zinc-binding motif.

## Data Availability

No new data were created or analyzed in this study. All data supporting the findings of this review are contained within the manuscript.

## Abbreviations

The following abbreviations are used in this manuscript:

EGFR	epidermal growth factor receptor
CDKs	cyclin-dependent kinases
BTK	Bruton tyrosine kinase
DNA	deoxyribonucleic acid
IARC	International Agency for Research on Cancer
SAR	Structure–activity relationship(s)
SRB	sulforhodamine B
MCF-7	human breast adenocarcinoma cell line
A549	human lung adenocarcinoma cell line
HCT-116	human colorectal carcinoma cell line
HT-29	human colorectal adenocarcinoma cell line
AsPC-1	human pancreatic adenocarcinoma cell line
SW1990	human pancreatic adenocarcinoma cell line
MRC-5	human fetal lung fibroblast cell line
PC-3	human prostate adenocarcinoma cell line
NMR	nuclear magnetic resonance
IR	infrared
ESI-MS	electrospray ionization mass spectrometry
HeLa	human cervical adenocarcinoma cell line
Caco-2	human colorectal adenocarcinoma cell line
MTT	3-(4,5-dimethylthiazol-2-yl)-2,5-diphenyltetrazolium bromide
SiHa	human cervical squamous cell carcinoma cell line
COLO205	human colorectal adenocarcinoma cell line
HepG2	human hepatocellular carcinoma cell line
HaCaT	human immortalized keratinocyte cell line
HER2	human epidermal growth factor receptor 2
SW480	human colorectal adenocarcinoma cell line
MDA-MB-231	human breast adenocarcinoma cell line
EGFRWT	wild-type epidermal growth factor receptor
EGFRL858R	L858R mutant epidermal growth factor receptor
RT-qPCR	reverse transcription quantitative polymerase chain reaction
S100A4	S100 calcium-binding protein A4
MMP-9	matrix metalloproteinase-9
AR	androgen receptor
LNCaP	Lymph node carcinoma of the prostate cell line
K562	human chronic myelogenous leukemia cell line
ABT-751	investigational tubulin polymerization inhibitor
CCK-8	Cell Counting Kit-8
B16F10	murine melanoma cell line
HEK	human embryonic kidney cells
A2780	human ovarian carcinoma cell line
CA-4	combretastatin A-4
RNA	ribonucleic acid
CMap	Connectivity Map
AKT	protein kinase B
VEGFR	vascular endothelial growth factor receptor
MAPK	mitogen-activated protein kinase
BRAF	B-Raf proto-oncogene serine/threonine kinase
JAK	Janus kinase
c-Met	hepatocyte growth factor receptor
PI3K/AKT/mTOR	phosphoinositide 3-kinase/protein kinase B/mammalian target of rapamycin
RAS/RAF/MEK/ERK	rat sarcoma/rapidly accelerated fibrosarcoma/mitogen-activated protein kinase kinase/extracellular signal-regulated kinase
JAK/STAT	Janus kinase/signal transducer and activator of transcription
CuAAC	copper-catalyzed azide-alkyne cycloaddition
LoVo	human colorectal adenocarcinoma cell line
LoVo/DX	doxorubicin-resistant LoVo cell line
p-EGFR	phosphorylated epidermal growth factor receptor
STAT3	signal transducer and activator of transcription 3
p-STAT3	phosphorylated signal transducer and activator of transcription 3
Bcl-2	B-cell lymphoma 2
BAX	BCL2-associated X protein
CDK-8	cyclin-dependent kinase 8
PDGFR	platelet-derived growth factor receptor
KIT	KIT proto-oncogene receptor tyrosine kinase
VEGFR-2	vascular endothelial growth factor receptor 2
CDK2	cyclin-dependent kinase 2
DU145	human prostate carcinoma cell line
WISH	human amniotic-derived epithelial cell line
WI-38	human fetal lung fibroblast cell line
HDAC	histone deacetylase
FDA	U.S. Food and Drug Administration
HDAC8	histone deacetylase 8
ZBG	zinc-binding group
